# Transfer Learning in Wastewater Treatment Plant Control Design: From Conventional to Long Short-Term Memory-Based Controllers

**DOI:** 10.3390/s21186315

**Published:** 2021-09-21

**Authors:** Ivan Pisa, Antoni Morell, Ramón Vilanova, Jose Lopez Vicario

**Affiliations:** 1 Wireless Information Networking (WIN) Group, Escola d’Enginyeria, Universitat Autònoma de Barcelona, 08193 Bellaterra, Spain; antoni.morell@uab.cat (A.M.); jose.vicario@uab.cat (J.L.V.); 2 Advanced Systems for Automation and Control (ASAC) Group, Escola d’Enginyeria, Universitat Autònoma de Barcelona, 08193 Bellaterra, Spain; ramon.vilanova@uab.cat

**Keywords:** control design, industrial control, transfer learning, WWTP

## Abstract

In the last decade, industrial environments have been experiencing a change in their control processes. It is more frequent that control strategies adopt Artificial Neural Networks (ANNs) to support control operations, or even as the main control structure. Thus, control structures can be directly obtained from input and output measurements without requiring a huge knowledge of the processes under control. However, ANNs have to be designed, implemented, and trained, which can become complex and time-demanding processes. This can be alleviated by means of Transfer Learning (TL) methodologies, where the knowledge obtained from a unique ANN is transferred to the remaining nets reducing the ANN design time. From the control viewpoint, the first ANN can be easily obtained and then transferred to the remaining control loops. In this manuscript, the application of TL methodologies to design and implement the control loops of a Wastewater Treatment Plant (WWTP) is analysed. Results show that the adoption of this TL-based methodology allows the development of new control loops without requiring a huge knowledge of the processes under control. Besides, a wide improvement in terms of the control performance with respect to conventional control structures is also obtained. For instance, results have shown that less oscillations in the tracking of desired set-points are produced by achieving improvements in the Integrated Absolute Error and Integrated Square Error which go from 40.17% to 94.29% and from 34.27% to 99.71%, respectively.

## 1. Introduction

Industrial environments are characterised by running complex and repetitive processes which are sometimes maintained over time. In that sense, control systems are adopted in order to ensure that these processes perform correctly [1]. Most of the times, the development of control strategies can become a complex and time-demanding task since a deep knowledge of the process under control is required. However, the incursion of the Industry 4.0 paradigm and Artificial Neural Network (ANNs) applications are changing the way we control and manage industrial environments. Their main aim is to provide the industries with solutions mainly based on measurements obtained from their systems [2]. Some of these solutions go from basic forecasting systems to more complex solutions, like predictive maintenance ([3], Chapter 9). However, one of the sectors where Industry 4.0 and ANNs are making the point corresponds to the industrial control ([3], Chapter 5). There, ANNs have been adopted for a wide range of tasks, such as the design of soft-sensors or the detection of malfunctions [4,5,6]. Not only this, but the industrial control domain is experiencing a change in its tendency: ANNs are used more and more as the main control structures than conventional controllers.

**Figure 1:**
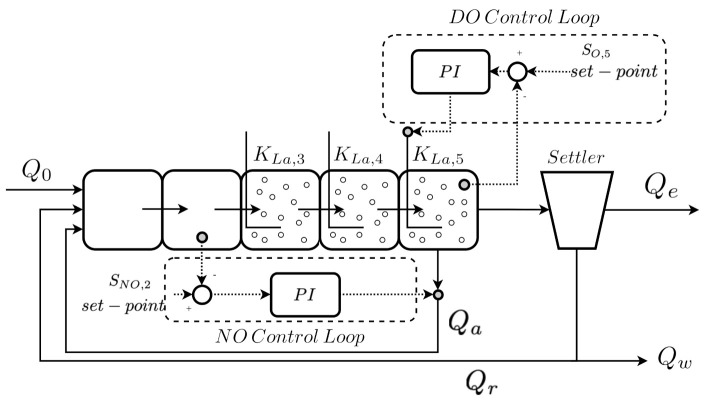
Benchmark Simulation Model No. 1 layout. $Q_{o}$, $Q_{a}$, $Q_{r}$, $Q_{e}$, and $Q_{w}$ are the influent, internal recycle, the external recycle, the effluent, and the wastage flow rates, respectively. Dotted lines correspond to control signals (measured concentrations, desired set-points and actuation signals), while solid lines correspond to process media.

One of the industrial sectors where this tendency is observed corresponds to the Wastewater Treatment Plants (WWTPs), which are characterised by running very complex processes where individual operations and actions can change the whole operation of the plant [7]. For that reason, a huge number of control loops are required in order to ensure that each individual operation is correctly performed. Proportional Integral (PI) controllers have mostly been considered as the default and basic controller strategy able to ensure correct WWTP behaviour [8]. However, a complete reduction of the pollutants present in the residual waters cannot be ensured. For that reason, more complex structures have been proposed in order to improve the control performance. Fuzzy and Model Predictive Controllers (MPCs) have been adopted in [9] as the main control strategy to avoid the effluent violations of a WWTP plant, whereas in [10], a hierarchical structure with fuzzy and MPC controllers has also been proposed to determine the control actuation, taking into account the weather and a variable set-point. In this case, the set-point adopted by the MPC controllers is determined by the fuzzy controller whose objective is to maintain the ammonium in the fifth reactor tank of a WWTP at a desired value (please observe Figure 1 to see the distribution of the tanks of a general-purpose WWTP). The problem observed with this kind of structure lies in the fact that they require a model which replicates the relationships between input and output measurements. Besides, most of the time, these relationships consist of non-linear relations which are difficult and tedious to model. This is where ANNs come in, since they are algorithms offering good performance when dealing with these kinds of relationships ([11], Chapter 6). The first approach consists of the adoption of ANNs as elements whose predictions are adopted by conventional control structures. For instance, the solution proposed in [10] has been improved in [12], where Long Short-Term Memory (LSTM) cells have been adopted to predict the WWTP effluent concentrations and determine when and which controller has to actuate. In other cases, neural networks have been considered to directly determine the optimal set-point values adopted by conventional controllers [13] or to implement a Reinforcement Learning (RL) module performing the same task [14]. Moreover, in the last few years, ANNs have been directly considered as the control strategy. In [15], neural networks have been considered to implement an Internal Model Controller (IMC) devoted to managing certain concentrations required in the pollutant reduction tasks performed in the WWTP. This also entails that the control actuation can be decoupled from the physical specifications of the environment [16,17].

The incursion of ANNs in the industrial control domain presents its own drawbacks that have to be taken into account [18]. The most important one consists in the fact that ANNs have to be designed and trained with amounts of data. This training process is devoted to determining the different hyperparameters of the ANNs, such as the numbers of hidden layers, neurons, learning rates, or even the topology of networks. This has to be performed for each ANN considered, either to complement a conventional controller (PI, MPC, Fuzzy), or to act as the controller as such. Besides, this training process can last hours or even days with regard to the network structure, the hyperparameters, and the amount of data, accordingly [19]. For that reason, transfer learning (TL) methods have been considered to alleviate these tasks.

TL was adopted from image classification tasks, where they were considered to obtain a good image classifier from predesigned and pretrained structures in a source domain [20]. Then, these pretrained structures were retrained with images of the target domain in what is called a fine-tuning process ([21], Chapter 6). In terms of industrial environments, TL was adopted in the design process of soft-sensors, where they are firstly designed and trained in a source domain where a huge number of measurements are available. TL techniques have been adopted mainly to design and implement soft-sensors in those harsh environments, showing a lack of measurements. In [22], TL techniques were considered to design a soft-sensor which would be deployed over a sulphur recovery unit. The problem there is that this environment shows a severe problem of data scarcity; therefore, a traditional ANN training process cannot be performed. To alleviate this, the authors proposed the adoption of TL to design and implement the soft-sensor in an environment without data scarcity problems (the source domain). Then, the obtained soft-sensor was transferred into the environment with the scarcity problem (the target domain) and fine-tuned to adapt its behaviour to this environment [22]. In our case, we propose the adoption of the Transfer Learning-based Control design approach to implement and design the complete control strategy of a general-purpose WWTP. The main idea is to substitute all the PI controllers by LSTM-based PI controllers, where only one is implemented while the others are obtained from transferred versions. The main point here is that instead of training and designing as many LSTM-based PIs as PI controllers, we will implement only a unique LSTM-based structure which will then be transferred into the remaining control loops. In that way, the design of the control loops can be eased at the same time its complexity is reduced. Now, efforts will be focused on designing a unique controller, which will be based on data instead of designing and tuning as many controllers as control loops. In this work, only two control loops have been designed and implemented following this approach. Thus, the benefit of this control approach is not as widely explored as it could be in a scenario where there exists multiple control loops, like in the petrochemical industry [4,23]. However, it has to be taken into account that this approach is mainly based on the adoption of ANNs, which are trained with amounts of data coming from the control loops. Therefore, data have to be accessible in order to adopt this approach; otherwise, the ANNs will not be properly trained, and consequently, the control loops will not act as they should.

This approach has firstly been conducted in [24], where a LSTM-based PI structure has been trained with data from a unique control loop and then transferred into the remaining control loop of a WWTP environment. Notwithstanding this, the structure proposed in [24] considers a unique LSTM cell which requires a total amount of 4 h of WWTP measurements in order to achieve a good control approach. Besides, neither the design of the LSTM-based PI, nor its fine-tuning process is carried out. Therefore, the control performance of the LSTM-based PI can be improved if it is fine-tuned with measurements coming from the target domain, that is, the control loop where the LSTM-based PI is transferred. For that reason, in this manuscript we will continue the work started in [24]. Here, we propose the fine-tuning process and we also analyse the benefits and losses of implementing the LSTM-based PI with data coming from different control loops. Moreover, a new LSTM-based PI structure able to manage the WWTP control loops without requiring 4 h of measurements will be proposed at the same time the control performance will be improved by means of the fine-tuning of this LSTM-based PI controller. Results will show that among all the LSTM-based PI, there exists one able to perform well in the different control loops. Thereby, the fine-tuning process of this LSTM-based controller and its control performance will also be analysed in this work. Besides, the speed-up of the design and implementation process will be explored and analysed in this manuscript as a function of the amount of time required to train the LSTM-based structures. The application where it is tested is specific, but the proposed design approach can be adopted in any kind of industrial environment where measurements are available. In summary, a TL-based design approach is proposed to implement the complete control strategy of a WWTP. The main contributions of this work can be summed up as:

- Conventional PI controllers will be substituted by LSTM-based structures able to improve the conventional controller performance.
- The required knowledge of the process under control will be reduced, since the LSTM-based structures only require input and output measurements of the conventional controllers. Besides, these measurements are easily obtained from a well-known WWTP digital framework: the Benchmark Simulation Model No. 1 [25].
- The design and implementation process of the LSTM-based structures will be sped up, since only a LSTM-based structure will be implemented from scratch. The remaining ones will be obtained through TL approaches.
- A fine-tuning process will be carried out to ensure that the control performance of the control loop is improved with respect to the conventional WWTP controller.

The structure of the manuscript is as follows. The work presented here is introduced in Section 1. The materials and methods adopted in this work are presented in Section 2, especially the Benchmark Simulation Model No. 1 (BSM1), a digital framework which models a general-purpose WWTP. In addition, the LSTM cells, as well as the TL principles are explained in this section. Then, the main contribution of this work, that is, the adoption of TL methods to design and implement the controllers of WWTP control loops are defined and explained in Section 3. The results of the exploratory analyses carried out are reflected in Section 4, while Section 5 concludes the paper.

## 2. Materials and Methods

### 2.1. Benchmark Simulation Model No. 1

The Transfer Learning-based Control Design approach proposed here is tested over the Benchmark Simulation Model No. 1 (BSM1). The BSM1 plant is a fictitious WWTP designed by using the engineering principles of an activated sludge process. It characterizes a medium-scale and general-purpose WWTP plant whose main objective is to reduce the nitrogen-derived pollutant products present in residual urban waters [25]. Besides, one of the major aims of BSM1 is to implement a digital framework where different control strategies can be designed and tested before being applied in the real environment. Thus, BSM1 is able to offer generality, easy comparison, and replicability of results in terms of the different control strategies devoted to maintaining certain pollutant components under certain levels or limits [8].

In such a context, BSM1 implements the Activated Sludge Model No. 1 (ASM1) which corresponds to a set of mathematical expressions describing the non-linear and highly complex biological and biochemical processes carried out inside the WWTP plant [26]. These processes mainly consist of the denitrification and nitrification processes where the nitrate and ammonia components are transformed into nitrogen and its derivate products [27]. Notwithstanding this, there are other Activated Sludge Models whose main aim is not only to model the processes carried out to reduce the nitrogen-derived pollutants, but also the phosphorus-derived ones. This is the case for the Activated Sludge Models No. 2, 2d, and 3 [28], which require some updates in the BSM1 framework in order to either consider the phosphorus removal processes like in the phosphorus removal BSM1 framework (BSM1-P), or the sludge treatment, like in the Benchmark Simulation Model No. 2 (BSM2) [29,30]. Nevertheless, the study of these behaviours, as well as the layout of these benchmarks is out of the scope of this work.

#### 2.1.1. BSM1 Layout

The BSM1 layout consists of a set of five reactor tanks and a settler placed just before spilling the clean water into the receiving waters (see Figure 1). The five reactor tanks, where the biological and biochemical processes described in the ASM1 model are carried out, are characterised by their aerated conditions: the first two are anoxic tanks (working with a lack of oxygen), whereas the last three work under aerated conditions [8]. They have a total volume of 6000 m${}^{3}$, 1000 m${}^{3}$ for each anoxic tank and 1333 m${}^{3}$ for each aerated tank. The settler has a total volume of 6000 m${}^{3}$. Thus, the total volume of BSM1 equals to 12,000 m${}^{3}$. Besides, the BSM1 framework has been designed to process an average influent flow rate equal to 18,446 m${}^{3}$/d and an average biodegradable chemical oxygen demand (COD) of 300 g/m${}^{3}$. This entails that the BSM1 retention time is equivalent to 14.4 h on average [8,31].

The influent data for municipal WWTP consists of time-series data of the flow and concentrations of the water quality parameters. These influent flow rates depend on many factors: the size of the catchment, the type of the sewer system, and the number of person equivalents, among others. For instance, influent profiles for a WWTP of 100,000 PE are available in [32]. They include dry, rainy, and stormy weather conditions. Besides, these are the usual ones considered when working with BSM1, and therefore the ones considered in the presented work. More information about the BSM1 influent flow and concentrations can be obtained in the BSM1 specifications [8]. Among these 15 variables, the ones of interest in this work are the ones related to the BSM1 default control strategies:

- Nitrate and nitrite nitrogen (NO) control loop: control loop in charge of controlling the nitrate and nitrite nitrogen concentration present in the second reactor tank ($S_{NO,2}$).
- Dissolved oxygen (DO) control loop: control loop in charge of managing the dissolved oxygen present in the fifth reactor tank ($S_{O,5}$).

In the case of the NO control loop, a proportional integral (PI) controller is proposed to manage the internal recycle flow rate ($Q_{a}$) in order to ensure that the $S_{NO,2}$ concentration is maintained at the default set-point (1 mg/L). The DO control loop considers another PI structure whose main aim is to maintain the $S_{O,5}$ concentration at the default set-point of 2 mg/L. This is performed by means of varying the oxygen transfer coefficient of the fifth reactor tank ($K_{La,5}$) accordingly to the measured $S_{O,5}$. In that sense, it is worth noting that the two default PI controllers provided in the BSM1 framework have already been tuned, that is, the proportional gain and the integral time parameters are predefined by the BSM1 designers. The control performance of these PI configurations is provided as a start and a baseline with which a new control structure can be compared. Moreover, we have considered the default control strategies, that is, their parameters have been left as the initial configuration proposed by the BSM1 designers.

#### 2.1.2. BSM1 Simulation and Evaluation Protocols

As previously stated, BSM1 has been widely considered as a general-purpose WWTP digital framework offering generality, easy replication, and comparison between different control strategies. In order to ensure a fair comparison in the control performance, BSM1 considers two kind of simulations: (i) a simulation where no variations are produced in the influent, and (ii) a simulation where daily influent and weather variations are produced. In that sense, four influent profiles considering 14 days of influent measurements are provided [25]:

- Constant influent: Influent profile showing constant influent concentrations and flow rates during 14 days.
- Dry influent: Influent profile showing daily variations of the influent concentrations and without any perturbation induced by weather changes.
- Rainy influent: Influent profile showing daily variations of the influent concentrations. Two large rainy perturbations are considered during days 9 and 10.
- Stormy influent: Influent profile showing daily variations of the influent concentrations. Two short but intense stormy perturbations are produced at days 8 and 11.

Thus, the kind of simulation is set accordingly to the influent profile considered in the simulation. However, the BSM1 model has to be previously initialised before performing the simulations. The initialisation process mainly consists in the stabilization of the BSM1 reactor tanks by means of simulating a total amount of 100 days of constant influent ([8], Section 3). Once the model is stabilised, one can perform the desired simulation. From the 14 days simulated, only the last seven days of the simulation, that is, day 7 to day 14 are considered in the performance computation ([8], Section 6). It is also worth noting that only dry, rainy, and stormy influent profiles are considered in this work.

BSM1 also considers its own performance metrics which ease the comparison process among control strategies. They can be divided into two main categories: the environmental metrics and the control ones. The environmental metrics are those showing the improvements achieved in terms of the pollutant reduction when a control strategy is considered instead of another one. Among the different metrics, the two most widely adopted ones are the Overall Cost Index (OCI) and the Effluent Quality Index (EQI). OCI is related to the costs generated in the pollutant reduction process, while the EQI can be understood as a metric telling how clean the water is [8,30]. Nevertheless, we will focus on the control metrics which do not have either an environmental nor a pollutant flavour. In our case, we are going to consider the Integrated Absolute (*IAE* ) and Integrated Squared Errors (*ISE*) between the measured variables and their corresponding set-points:

(1) $$IAE=\int_{t=7^{th}\;day}^{t=14^{th}\;day}\left|r(t)-y(t)\right|\;dt$$

(2) $$ISE=\int_{t=7^{th}\;day}^{t=14^{th}\;day}{\left(r(t)-y(t)\right)}^{2}\;dt,$$

where r(t) corresponds to the desired set-point, and y(t) to the measured concentration. In this case, $y\left(t\right)=\left\{S_{NO,2}\left(t\right),\;S_{O,5}\left(t\right)\right\}$. Notice that only the control metrics are considered due to the fact that this work is mainly focused on the adoption of transfer learning approaches and ANNs to ease and speed up the design and implementation of the control strategies.

### 2.2. Long Short-Term Memory Cells

**Figure 2:**
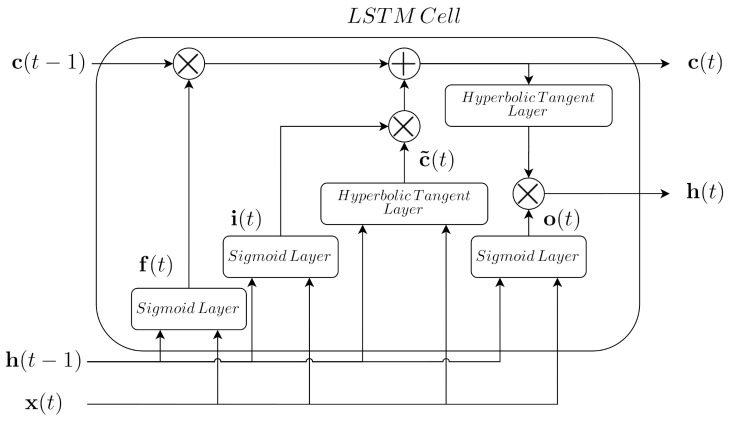
LSTM cell internal structure.

The ANN-based PI controller adopted in this work is mainly based on Long Short-Term Memory (LSTM) cells. They correspond to a type of gated networks which are characterised by their good performance when dealing with time-series signals ([11], Chapter 10). This is possible thanks to the gates that each LSTM cell implements: (i) three sigmoid activation layers, the input gate (i(t)), the forget gate (f(t)), and the output gate (o(t)), and (ii) one hyperbolic tangent layer, the state gate ($\tilde{c}\left(t\right)$) (see Figure 2).

In terms of data, the LSTM cell considers the input data (x(t)) and the output data (h(t)) vectors. Accordingly to them, the forget gate determines the amount of the cell state information that has to be deleted:

(3) $$f\left(t\right)=\sigma (W_{f}\cdot x\left(t\right)+U_{f}\cdot h\left(t-1\right)+b_{f}\left(t\right)).$$

Then, the input and state gates determine the new information to be stored in the cell state:

(4) $$i\left(t\right)=\sigma (W_{i}\cdot x\left(t\right)+U_{i}\cdot h\left(t-1\right)+b_{i}\left(t\right))$$

(5) $$\tilde{c}\left(t\right)=tanh\left(W_{c}\cdot x\left(t\right)+U_{c}\cdot h\left(t-1\right)+b_{c}\left(t\right)\right)$$

(6) $$c\left(t\right)=f\left(t\right)\circ c\left(t-1\right)+i\left(t\right)\circ \tilde{c}\left(t\right).$$

Finally, the output data of the LSTM cell is computed as a function of the input and previous output, as well as the outcome of the output gate:

(7) $$o\left(t\right)=\sigma (W_{o}\cdot x\left(t\right)+U_{o}\cdot h\left(t-1\right)+b_{o}\left(t\right))$$

(8) h(t)=o(t)∘tanh(c(t)).

Notice that $W_{x}$ and $U_{x}$ are the weights of the different gates modifying the input and output data vectors, respectively. $b_{x}$ are the biases of the different gates. Finally, ∘ is the Hadamard product between two matrices. σ and tanh are the sigmoid and hyperbolic tangent activation functions, respectively. If more information about LSTM cells and their behaviour is required, readers are referred to ([11], Section 10.10).

### 2.3. Transfer Learning

The main contributions of this work are mainly focused on the adoption of Transfer Learning (TL) techniques to ease and speed up the control design in industrial environments, especially in the WWTPs. In that sense, TL consists of transferring the knowledge obtained in the training process of an ANN structure into another one. For instance, TL techniques have been widely adopted in the design and implementation of image classifiers among others [20]. One clear example is shown in ([21], Chapter 6), where the Inception model, a general-purpose image classifier, is adopted to develop a dog breed classifier. This new classifier is implemented with the Inception classifier without the last layer plus three new convolutional layers connected to the output of the penultimate Inception layer. Therefore, the dog breed classification performance will be derived from the Inception classification one and a new retraining process where a new set of dog breed pictures is considered ([21], Chapter 6). This shows that TL techniques can be considered as techniques which not only obtain ANN models performing well from a source model, but also speed up their designing process since the knowledge of the source model is shared with the new ones ([21], Chapter 4).

In that sense, TL techniques can be categorised into three classes as a function of the data availability in the source and target domains or scenarios ([21] Chapter 4, [22]):

- Inductive Transfer Learning: In inductive transfer learning, the source and target domains do not show data scarcity problems. Therefore, the transfer model can be designed and firstly trained in the source domain and then fine-tuned in the target domain in order to adapt its behaviour to its final application.
- Transductive Transfer Learning: Transductive transfer learning is characterised by the necessity of retraining the transferred model every time a new set of labelled data is available in the target domain. This is motivated by the fact that at the first moment, the target domain has no labelled data.
- Unsupervised Transfer Learning: Unsupervised transfer learning is characterised by the fact that there is no available data neither in the source domain, nor in the target one. Thus, this technique is mainly focused on solving unsupervised tasks like dimensionality reduction.

In our case, we are faced with an Inductive Transfer Learning task, since the source and target domains do not show a data scarcity problem. Here, the source domain consists in the $S_{O,5}$ control loop (DO control loop) or the $S_{NO,2}$ control loop (NO control loop) depending on the base ANN-based controller being implemented. In that sense, if the DO control loop is considered as the source domain, the NO control loop will be considered as the target domain and vice versa.

### 2.4. Modelling

Three different tools have been considered in this work to implement and test the proposed Transfer Learning-based Control Design approach. They correspond to Simulink and Python. Simulink was adopted due to the fact that the BSM1 model is completely deployed over this simulator. Simulink version 10.1 running over Matlab R2020b was considered. Moreover, all the ANNs involved in the proposed approach are also deployed over the BSM1 model in order to test their behaviour. Thus, they are also implemented in Simulink. In that sense, the ANNs, and especially the LSTM cells have been designed and trained by adopting Python 3.6 with three open-source libraries and a NVIDIA GeForce RTX 2080 Titan GPU memory, which is considered to speed up the LSTM training process:

- NumPy (1.18.1) [33]: library providing a huge amount of tools and operations involving vectors and matrices.
- Scikit-Learn (0.22.1) [34]: library providing most of the functions considered in data preprocessing, cross-validation, and evaluation processes.
- Tensorflow (1.14.0) [35]: library providing lots of ANN structures and techniques. It also implements the Keras API, which offers predefined ANN structures, optimizers, cost functions, or training algorithms. Therefore, nearly any ANN structure can be designed by means of concatenating different predefined Keras structures.

## 3. TL-Based Control Design

As it has been stated, one of the problems in industrial control is related to the conception and design of the control loop. Most of the times, the design of the controllers can become a tedious and time-consuming process since one has to determine the topology of the controller to be used, as well as the plant or process it is going to manage. In that sense, ANNs have arisen as a possible solution able to alleviate this. They only require pairs of input and output data of the process to be controlled [15]. However, this has its own drawbacks: ANNs have to be correctly trained and designed if a good control performance is required. This can become a time-demanding and computationally expensive process if there are a lot of control loops to design.

**Figure 3:**
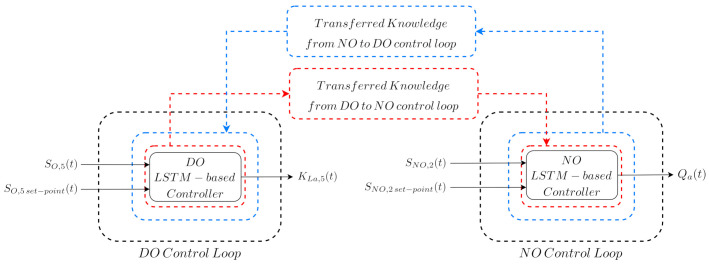
Graphical description of the TL-based Control Design approach. Notice that DO refers to the Dissolved Oxygen ($S_{O,5}$) control loop, whilst NO refers to the nitrate and nitrite ($S_{NO,2}$) control loop.

For that reason and to alleviate this issue, we propose in this work the TL-based Control Design approach, which is focused on designing and implementing the control strategies of a general purpose WWTP. In this case, the TL-based Control Design approach consists in two stages: (i) the LSTM-based controller, where the design and training of an ANN-based controller is carried out, and (ii) the Control Knowledge Transfer approach, where the transfer of the controller knowledge into the different industrial control loops is performed. The first stage is mainly based on designing an ANN able to manage the signals considered in the control of the industrial process. To achieve this, the proposed ANN-based controller predicts the corresponding actuation signal accordingly to its input measurements, that is, the measured value and its set-point. In our case, the signals involved in the control loops correspond to either the $S_{O,5}$ or the $S_{NO,2}$ concentrations, and their respective actuation signals, the $K_{La,5}$ or $Q_{a}$. Besides, the ANN-based controller will be implemented with LSTM cells due to their good performance when dealing with time-series signals, such as the ones obtained from the BSM1 framework. ([11] Section 10.10, [8]). The second stage is mainly focused on transferring the knowledge of the proposed LSTM-based controller into the other control loops. In this case, the LSTM-based controller is considered as the baseline strategy to be transferred (see Figure 3). Thus, the objective is to design only a LSTM-based controller instead of as many LSTM-based structures as control loops present in the WWTP. Then, its knowledge will be transferred into the remaining loops.

It is also important to notice that this transfer approach can be adopted in any industrial scenario. However, there is a requirement that has to be fulfilled; the TL-based Control Design can only be applied among control loops sharing the same control objective. This is motivated by the fact that the ANN-based structure trained in the source control loop will learn how to generate an actuation signal from the controlled ones with the objective of performing certain tasks, for instance, the tracking of a given set-point. Then, the knowledge of this ANN-based structure will be transferred into the target one, which should have the same objective. Otherwise, the target structure would generate actuation signals which do not fulfil the control objective. In the case of this work, the control objective is clear, where both the DO and the NO control loops are designed to track the given set-points regardless of the fact that the involved signals show different values and dynamics [8,25].

Once the knowledge of the LSTM-based control structure is transferred, the control performance of the LSTM-based controller can be adjusted through a fine-tuning process which consists in a retraining of the LSTM-based structure. However, this fine-tuning process is different from the usual fine-tuning processes performed in the usual applications of transfer learning, that is, the development of image classifiers. There, the data considered to carry out the fine-tuning process consist in a set of new images where the labels are intrinsically obtained from the same images. Taking up the dog breed classifier, the TL fine-tuning process is performed to the Inception structure with images of different dog breeds where the labels are obviously clear. When talking about industrial processes, the situation completely changes. The new measurements have to be obtained from the control loop where the LSTM-based controller is going to be transferred. In addition, the knowledge about how to control this loop has to also be obtained. For that reason, the data required to perform the fine-tuning processes have to be obtained by means of simulating the behaviour of the industrial process when an existing and conventional controller is applied. If not, the LSTM-based controller will not be able to offer a good control performance.

In this manuscript, two LSTM-based controllers will replicate the behaviour of the default WWTP PIs since they are the ones present in the BSM1 digital framework [8]. This is motivated by the fact that the LSTM-based PI controller can be obtained with data from the DO control loop (DO LSTM-based PI) and then transferred into the NO control loop, or it can be designed considering measurements from the NO control loop (NO LSTM-based PI) and then transferred into the DO loop. From these two controllers, the one offering the best control performance in both control loops will be fine-tuned.

### 3.1. LSTM-Based PI

**Figure 4:**
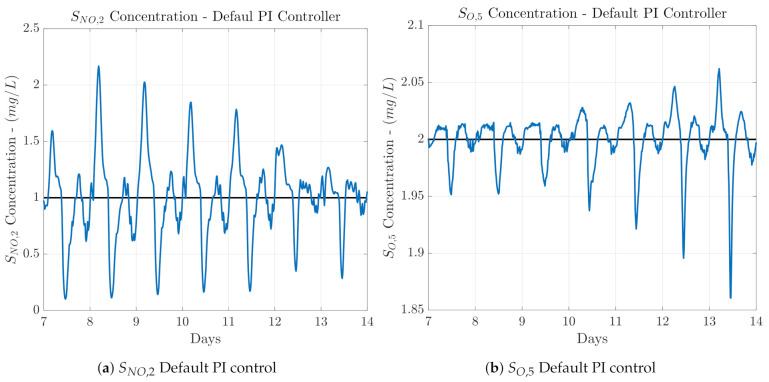
Control performance when the default PI controllers are adopted. Notice that the worst performance is offered by the $S_{NO,2}$ default PI controller.

As it has been previously stated, the controller proposed in the TL-based Control Design approach consists in a LSTM-based controller which will act as a PI controller managing either the $S_{O,5}$, or the $S_{NO,2}$ concentrations. Hence, two LSTM-based PI candidates are proposed since there exists two control loops, the DO and the NO control loop. For that reason, we will analyse the control performance of each one in order to determine which LSTM-based PI will be transferred and fine-tuned. The first LSTM-based PI controller corresponds to the DO LSTM-based PI, which is derived from the PI managing the $S_{O,5}$ concentration, while the second one corresponds to the NO LSTM-based PI. It is derived from measurements of the default PI managing the $S_{NO,2}$. Before designing and training the two LSTM-based PIs, one can guess which one will offer the best control performance. If the control performance of the default PI controllers is taken into account (see Figure 4), one can observe that the best PI corresponds to the one managing the $S_{O,5}$, since it is able to maintain the $S_{O}$ concentration at the desired value (2 mg/L). On the other hand, the PI managing the $S_{NO,2}$ is not able to maintain the desired set-point. Thereby, the control performance of the LSTM-based ones will be similar to the default PI controller from which the data were obtained. In other words, the better the conventional controller performance, the better the LSTM-based one.

The DO LSTM-based PI and the NO LSTM-based PI structures are obtained by means of a grid search method where different LSTM-based structures are trained with the same set of measurements. The efforts of the grid search are focused on determining the number of LSTM cells, feedforward layers, and hidden neurons per layer of the LSTM-based structure. Then, the LSTM structure offering the best prediction performance without committing overfitting is the one considered as the main structure in which the LSTM-based PI is based on. In that sense, the grid search is performed instead of finding the parameters characterising the PI controller, that is, the integral time and the proportional gain [36]. This means that a deep knowledge of the process under control is not required. Only pairs of input and output measurements of the existing default PI controllers are needed. To obtain them, a complete year of randomly distributed weather profiles has been simulated in order to achieve a good control performance regardless of the weather conditions. From all the available measurements, the input and output measurements of the LSTM-based PI controller will be determined accordingly to the control loop they will manage:

- DO Control loop: the measurements involved in the DO control loop are the dissolved oxygen ($S_{O,5}$), its desired set-point ($S_{O,5_{set-point}}$), and the oxygen transfer coefficient of the fifth reactor tank of the WWTP ($K_{La,5}$).
- NO Control loop: the measurements involved in the NO control loop are the nitrate and nitrite nitrogen ($S_{NO,2}$), its desired set-point ($S_{NO,2_{set-point}}$) and the internal recirculation flow of the WWTP ($Q_{a}$).

**Figure 5:**
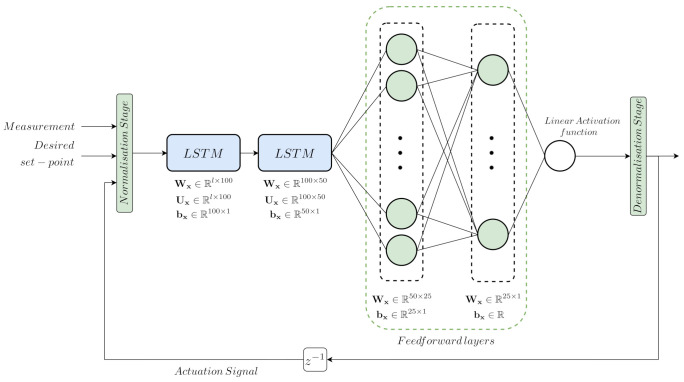
LSTM-based net considered in the LSTM-based Controller. *l* corresponds to the number of inputs, which in this case is set to three measurements: the measured concentration of interest, $S_{O,5}\left(t\right)$ or $S_{NO,2}$, its set-point, $S_{O,5_{set-point}}\left(t\right)$ or $S_{NO,2_{set-point}}\left(t\right)$, and the actuation variable, $K_{La,5}\left(t-1\right)$ or $Q_{a}\left(t-1\right)$.

These measurements are the ones considered to carry out the grid search method devoted to determining the LSTM-based PI structures. Each one of these measurements is split into three different sets: 70% of the measurements to train the different LSTM-based net configurations, 15% to validate them, and the remaining 15% to test the structures. The grid search process has been carried out adopting the Adam optimizer ([11], Sections 6.5 and 8.5.3) and a total amount of 500 epochs. The initial learning rate value has been set to $1\times 10^{-3}$, however, it is reduced along the process. In addition, LSTM nets are also known to suffer overfitting problems, where they memorise the input and output measurements instead of deriving a model from them ([11], Chapter 7). To avoid this problem, the L2 parameter regularisation technique and early stopping method are considered. The L2 parameter regularisation consists in the addition of extra penalty to the weights of the corresponding layer ([11], Section 7.1.1). This extra penalty is known as the weight decay parameter, which in this case has been set to $5\times 10^{-4}$. On the other hand, early stopping acts as a technique which stops the training process when the validation performance changes its tendency with respect to the training one ([11], Section 7.8). Here, the important point corresponds to the early patience, which determines the amount of epochs that this change of tendency is allowed. In this work, we consider an early patience of five epochs, understanding an epoch as a complete pass over the training dataset ([37], Chapter 2). Both LSTM-based controllers consider the same LSTM-based structure (see Figure 5) which mainly consists in two LSTM cells devoted to extracting and obtaining information from the time correlation between measurements and two feedforward layers which will transform this information into the desired output. Moreover, each structure considers Normalisation and Denormalisation stages in charge of normalising the input measurements towards zero mean and unity variance, and to take them into its natural range, respectively. These two stages are needed since the range of the measurements involved in the control loops are quite different: the mean of the measurements involved in the DO control loop equal to 1.9752 and 144.68 for the $S_{O,5}$ and the $K_{La,5}$, respectively. In the case of the NO loop, the mean values of the variables involved in the control are equal to 0.9937 and $2.1802\times 10^{4}$ for the $S_{NO,2}$ and $Q_{a}$ measurements. As a summary, the DO LSTM-based and NO LSTM-based structures are as follows:

- DO LSTM-based PI
  – Input measurements: the dissolved oxygen in the fifth reactor tank ($S_{O,5}\left(t\right)$) and its desired set-point ($S_{O,5_{set-point}}\left(t\right)$). Besides, the DO LSTM-based net considers the Nonlinear Autoregressive Exogenous principle (NARX) where the output predicted by the net will be considered as an extra input. This extra input provides the LSTM-based structure with information about its performance in the prediction process [38], thus it will be able to correct its predictions as a function of this extra input. In this case, the extra input corresponds to the previously computed actuator signal ($K_{La,5}\left(t-1\right)$).
  – Normalisation Stage: stage devoted to normalising the input measurements towards zero mean and unity variance.
  – LSTM-based Net: main part of the LSTM-based Controller. It consists of two LSTM cells with 100 and 50 hidden neurons and two feed forward layers with 50 and 25 hidden neurons, respectively.
  – Denormalisation Stage: stage devoted to denormalising the actuation signal (DO LSTM-based Net output) towards its real range of values.
  – Output: the actuation signal which corresponds to the oxygen transfer coefficient of the fifth reactor tank ($K_{La,5}\left(t\right)$).
- NO LSTM-based PI
  – Input measurements: the nitrate and nitrite nitrogen in the second reactor tank ($S_{NO,2}\left(t\right)$) and its desired set-point ($S_{NO,2_{set-point}}\left(t\right)$). As it happens with the DO LSTM-based PI, the NO LSTM-based controller also considers the NARX principle. In this case, the extra input corresponds to the previously computed actuator signal ($Q_{a}\left(t-1\right)$).
  – Normalisation Stage: stage devoted to normalising the input measurements towards zero mean and unity variance.
  – LSTM-based Net: main part of the LSTM-based Controller. It consists of two LSTM cells with 100 and 50 hidden neurons and two feed forward layers with 50 and 25 hidden neurons, respectively.
  – Denormalisation Stage: stage devoted to denormalising the actuation signal (DO LSTM-based Net output) towards its real range of values.
  – Output: the actuation signal which corresponds to the WWTP internal recirculation flow rate ($Q_{a}\left(t\right)$).

The prediction performance of both structures has been computed in terms of the difference between the predicted actuation variables and the expected ones (remember that the DO LSTM-based PI predicts the $K_{La,5})$, whereas the NO one predicts the $Q_{a}$. Five metrics are adopted, the Root Mean Squared Error (RMSE), the Mean Absolute Error (MAE), the Mean Average Percentage Error (MAPE) the determination coefficient ($R^{2}$), and the training time [39]. The RMSE and the MAE tell us how the prediction errors are, that is, if the predictions are close to the expected measurements or not. However, they are absolute metrics in terms of how they do not tell us how big or small these errors are. For that reason, we consider the MAPE, which compares the errors with respect to the expected value. $R^{2}$ is considered to determine the correlation between the predicted and expected measurements. Finally, the training time is considered to determine the amount of time to train unique network. Notice that all the prediction metrics are computed considering normalised values, with the exception of the MAPE in order to avoid divisions by zero and the training time. In that sense, the results show that the proposed LSTM-based structures are able to offer a good prediction performance (see Table 1) since both structures yield low RMSE, MAE and MAPE values at the same time they offer a $R^{2}$ nearly equal to 1. Therefore, it is corroborated that these structures can be used to implement PI controllers which are mainly based on data.

### 3.2. Control Knowledge Transfer Approach

The Control Knowledge Transfer approach corresponds to the stage of the TL-based Control Design devoted to transferring the knowledge of the LSTM-based PI structures of one WWTP control loop into the other. The adoption of this stage is motivated by the fact that we looked for the ease and speed-up of the controller design and implementation process, respectively.

In this manuscript, three different TL approaches are considered to achieve the transfer of the control knowledge between control loops. Two of them are considered to determine which controller, the DO or the NO LSTM-based PI, has to be transferred and then fine-tuned. The third approach mainly consists in the adoption of the controller showing the best performance in the source and target domains and its fine-tuning to adapt its behaviour to the dynamics of the target domain, the control loop where it has been transferred. As a summary, the three considered control approaches are:

- Transfer Learning from DO to NO
  The DO LSTM-based PI structure is transferred directly from the DO to the NO control loop. Here, it is important to notice that the structure is not fine-tuned, that is, the LSTM-based PI controller has been trained to manage the $S_{O,5}$ concentration. Besides, only the normalisation and denormalisation stages are adapted to the NO control loop measurements.
- Transfer Learning from NO to DO
  The NO LSTM-based PI structure is directly transferred from the NO to the DO control loop without performing any change, neither in its structure, nor in its weights and biases. Thus, the knowledge on how to manage $S_{NO,2}$ concentration is transferred into the DO control loop. The unique change performed in this transfer approach corresponds to the normalisation and denormalisation stages. They have been adapted to normalise and denormalise the measurements coming from the NO control loop instead of the DO control loop. Following this, the NO LSTM-based PI will be at least equal to the default PI managing the $S_{NO,2}$ concentration, that is, the NO control loop PI. If Figure 4 is taken into account, one can assure that the NO LSTM-based PI controller will not offer such a good control performance as the DO LSTM-based PI derived from the DO control loop.
- LSTM-based controller Fine-tuning & Transfer
  This transfer approach is the most important one since it corresponds to the transfer method performing a fine-tuning process and therefore, adapting the behaviour of the transferred controller to the target domain dynamics. In other words, in this transfer learning approach the LSTM-based controller yielding the best control performance between the Transfer Learning from DO to NO and the Transfer Learning from NO to DO will be considered as the candidate to be fine-tuned. Results in Section 4 show that the best control performance is offered by the DO LSTM-based PI. For that reason, this is the LSTM-based controller considered in the fine-tuning process. Nevertheless, this choice can be done at the very beginning if the performance offered by the conventional PI structures is considered (see Figure 4).
  In terms of the three TL classes, the LSTM-based controller Fine-tuning and Transfer approach consists in an inductive transfer learning task: data from the source domain, the DO control loop, is considered to firstly obtain the DO LSTM-based PI structure. Then it is fine-tuned (retrained) with data coming from the PI controlling the target domain, the NO control loop. In other words, the default $S_{NO,2}$ controller whose performance is observed in Figure 4a has been considered to perform the fine-tuning process of the DO LSTM-based PI controller. Thus, the obtained controller, the fine-tuned DO LSTM-based PI (FTDO LSTM-based PI) will know how to correctly manage the desired variable, but adapted to the NO control loop. This clearly shows that an existing controller managing the target control loop is compulsory to obtain the measurements considered in the fine-tuning process. This differs from traditional and conventional TL applications, where labelled data are available.
  The main point here is that in the fine-tuning process not all the layers of the DO LSTM-based PI controller will be retrained with measurements of the target domain: the weights of the two LSTM cells are blocked whilst the weights and biases of the two feedforward layers (see Figure 5) are modified in the fine-tuning process. The LSTM cells are the ones that are blocked since they are the layers gathering the information about the time-dependence between measurements. The feedforward layers mainly take this information to adapt the output of the controller to the desired control loop. For that reason, these are the layers which will be retrained just to adapt the outcomes of the LSTM layers to the new domain.
  The measurements of the target domain are again obtained by performing a whole-year simulation of the BSM1 behaviour when the three weather profiles, dry, rainy, and stormy, are randomly distributed. The weights and biases of the two retrained feedforward layers are obtained considering the same training parameters as in the case of the DO LSTM-based PI training process: initial learning rate equals to $1\times 10^{-3}$, the weight decay equals to $5\times 10^{-4}$ and the early patience is set to 5 epochs.

## 4. Results

### 4.1. TL-Based Control Design Results

The performance of the TL-based Control Design approach is determined by means of analysing the control performance of each one of the proposed TL approaches: (i) the Transfer Learning from DO to NO, (ii) the Transfer Learning from NO to DO, and (iii) the LSTM-based controller Fine-tuning and Transfer. In that sense, the two first results will determine which controller, the DO LSTM-based PI or the NO LSTM-based PI, is performing better in both control loops when no fine-tuning process is carried out. Finally, the one performing better is fine-tuned and its control performance is computed in the last TL approach. Results will show which is the best option not only to obtain a complete and good control approach mainly based on data, but also to speed-up the design process of the complete WWTP control strategy.

The control performance has been computed in terms of fix and variable set-points in order to determine if the TL-based Control Design approach is suitable for both types of set-points. Fix set-points are considered since the default control strategy considers them in order to assure that the nitrification and denitrification processes, the ones performing the pollutant reduction task, are correctly performed [8,27]. They have been set to 2 mg/L and 1 mg/L for the $S_{O,5}$ and $S_{NO,2}$ control loops, respectively. Notwithstanding, variable set-points are the ones of most interest since most of the times the set-points are computed by means of other control strategies or are varied in order to optimise the pollutant reduction process [10,14,40,41]. In this case, the variable DO set-point has been computed accordingly to the Fuzzy Logic adopted in [10], where the Fuzzy controller is considered to determine the $S_{O,5}$ set-point generating the lower $S_{NH,5}$. Moreover, the three different BSM1 weather profiles have also been simulated to determine if the control design approach can be considered regardless of the weather conditions.

### 4.2. Transfer Learning from DO to NO

The first computed control performance corresponds to the situation where the DO LSTM-based PI is obtained with data from the DO control loop. Then, it is transferred into the NO control loop without performing any fine-tuning process. Results are shown in Table 2, where the first important effect that one can notice is that the control performance in the DO control loop, that is, in the management of $S_{O,5}$, is even better than the control offered by the default PI. This effect is motivated by two situations: (i) the fact that the DO LSTM-based PI has been trained through the simulation of the control strategy when random variations in the set-point are provided, and (ii) the NARX principle which provides the LSTM-based structure with information about the previous predicted outcomes. Thus, the LSTM-based structure has learnt how to correct variations present either in the set-point, or in the measured concentration.

In terms of the *IAE* and *ISE* metrics, one can observe that they are improved with respect to the default PI control performance when a fixed set-point is considered. In addition, these improvements are achieved regardless of the weather profile. In other words, the *IAE* and *ISE* values were improved by around a 95.98% and a 99.84% in average with respect to the default PI controller, respectively. For instance, the highest *IAE* improvement is achieved when the stormy influent profile is simulated. The *IAE* offered by the default $S_{O,5}$ PI controller is equivalent to 0.158, while it is reduced until 0.006 when the DO LSTM-based PI is considered. This entails that the difference between the measured and the $S_{O,5}$ controller is minimal. In terms of the *ISE*, the highest improvement is obtained when the dry weather is simulated. The achieved improvement equals to 99.86% with respect to the default PI controller. Notwithstanding, this improvement equals to 99.84% and 99.82% when the rainy and stormy weathers are considered, respectively. These results show that the DO LSTM-based PI is able to be highly improved when a fixed set-point is considered. However, the important results are the ones obtained when a variable set-point is considered, since it corresponds to the most frequently adopted set-point topology.

**Figure 6:**
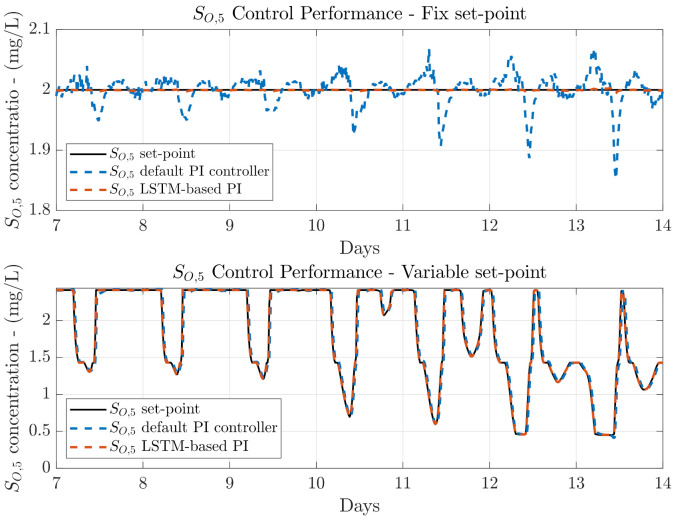
Control performance for the DO control loop when the LSTM-based PI is considered.

In such a context, the same effect is observed when a $S_{O,5}$ variable set-point is considered. In this case, the average improvement in terms of the *IAE* and *ISE* equals to 91.67% for the *IAE* and a 97.77% for the *ISE*. Now, the highest improvement is achieved when the dry weather is considered: the *IAE* and the *ISE* are improved by 92.97% and 98.54% with respect to the default PI controller performance. This is motivated by the fact that rainy and stormy influents are derived from the dry weather where the rainy and stormy episodes are included. For that reason, the LSTM-based structure has more often observed the effects of the PI controlling the $S_{O,5}$ when dry episodes are observed rather than stormy or rainy ones. In addition, the control performance clearly shows that the DO LSTM-based PI controller can be adopted as the main controller in the DO control loop (see Figure 6). As it is observed, the output of the controller is much closer to the given set-point of 2 mg/L than the default PI output.

**Figure 7:**
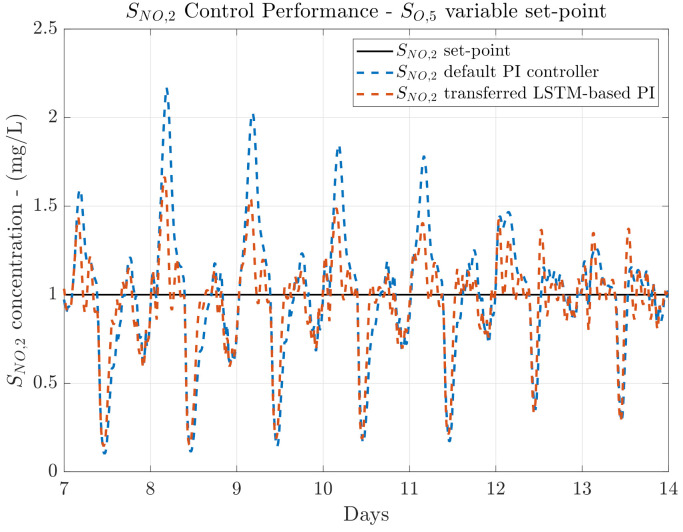
Control performance for the NO control loop when the LSTM-based PI derived from the DO control loop is transferred into it.

However, the most important point is to determine the control performance of the NO control loop, since in this case the DO LSTM-based PI is directly transferred into the NO control loop. The changes performed in the control structure correspond to the normalisation and denormalisation stages, which have been adapted to the range of values involved in the control of $S_{NO,2}$. Results show that the control performance of the DO LSTM-based PI controller can be improved by, on average, 33.07% and 42.94% in the case of the *IAE* and the *ISE*, respectively, when it is managing the $S_{NO,2}$ and considering a fixed set-point. For instance, the highest improvement with respect to the default PI structure is achieved when the stormy weather is considered. The *IAE* and *ISE* obtained in such a situation equal to 1.033 and 0.357, respectively, which in percentage values equal to an improvement of a 44.88% and a 63.46% for the *IAE* and *ISE* respectively. At the same time, this represents a reduction of the *IAE* and *ISE* improvement of 51.32 and 36.36 percentage points with respect to the improvement achieved when the DO LSTM-based PI is managing the $S_{O,5}$. This is clearly motivated by the fact that the DO LSTM-based PI is designed to offer its best performance when managing the DO control loop. When a $S_{O,5}$ variable set-point is chosen, one can observe that the average improvements in the NO control loop and in terms of the *IAE* and *ISE* are equal to 26.19% and 37.27%, respectively, being the dry weather the one showing the highest improvement (see Figure 7). The *IAE* values go from 1.792 to 1.271 while the *ISE* values go from 0.858 to 0.503, respectively.

These results show that the DO LSTM-based PI controller is able to improve the default PI controllers performance. For that reason, it is considered as a candidate to be fine-tuned in order to adapt its behaviour to the $S_{NO,2}$ control management and therefore, achieve a better improvement in the management of this loop.

### 4.3. Transfer Learning from NO to DO

Before performing the fine-tuning process, the control performance of the NO LSTM-based PI is also computed to determine its behaviour when managing the NO control loop (its source domain) and its performance when managing the DO loop (its target domain). Results are shown in Table 3 where at first sight it is clearly observed that the *IAE* and *ISE* metrics are improved with respect to the default $S_{NO,2}$ PI controller. When a $S_{O,5}$ fix set-point is considered, the NO control loop *IAE* is improved in average a 24.32% while the corresponding *ISE* is improved around a 39.03% in average. Both with respect to the default NO control loop PI controller. The *ISE* improvement shows that the proposed NO LSTM-based PI controller, which has been derived from the NO control loop, is able to reduce the highest errors between the measured $S_{NO,2}$ and its set-point, with respect to the default PI controller. However, the control performance can be still improved since the improvement achieved in terms of the *IAE* error is still low. For instance, the best improvement is observed when the stormy weather is considered. There, the obtained *IAE* goes from 1.874 to 1.360, whereas the *ISE* goes from 0.977 to 0.543. These values represent an improvement around a 27.43% and a 44.42% when the obtained *IAE* and *ISE* values are compared to the default PI control metrics. In terms of the $S_{O,5}$ control performance, the transferred NO LSTM-based PI shows that the *IAE* performance is degraded instead of improved. For instance, when the NO LSTM-based PI is adopted, the *IAE* is increased from 0.148 to 0.158 when the dry weather is considered. This effect is motivated by the fact that the default PI of the NO control loop is not offering such a good control performance as the default PI of the DO control loop. Thus, the control performance will not be improved if data from the NO control loop is obtained to derived the NO LSTM-based PI and then transfer it into the DO control loop.

**Figure 8:**
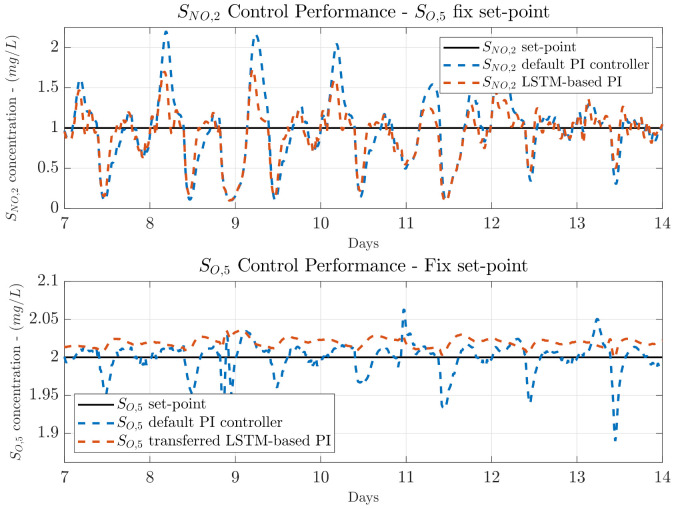
Control performance for the NO and DO control loops when the stormy weather is considered. The LSTM-based PI managing the DO control loop is derived from the NO control loop and transferred into the DO one.

Visually, one can observe that the $S_{NO,2}$ control performance is slightly improved with respect to the default PI (see Figure 8). The peaks of $S_{NO,2}$ concentration are reduced, however, the desired set-point is not achieved. In terms of the $S_{O,5}$, the control performance is even slightly degraded with respect to the default PI controller. As it can be observed, the measured $S_{O,5}$ does not show variations as the default PI controller, however, there exists an offset which produces the *IAE* increment. For that reason, the *ISE* metric in terms of the $S_{O,5}$ is still reduced, it now equals to 0.004 in average instead of 0.007. Notice that the *ISE* tells if there exists a huge difference between the measured and the desired concentration, whereas the *IAE* tells if the difference is maintained over time.

When a variable set-point is considered, one can observe that the control performance is only improved in terms of the NO control loop. The *IAE* and *ISE* metrics are improved in averages with respect to the default PI controller of 27.56% and 42.94%, respectively. In terms of the DO control loop performance, results show that transferring the NO LSTM-based PI controller derived from the NO loop into the DO loop is not an option, since all the control metrics are degraded. For instance, the *IAE* and *ISE* metrics are nearly doubled with respect to the default PI controller when the stormy weather profile is simulated. These results entail that the NO LSTM-based PI cannot be considered as a candidate to be fine-tuned since it does not improve the control performance of target domain at the same time that the improvement achieved in the source domain is much lower than the one achieved by the DO LSTM-based PI. In addition, this also corroborates one of the main ideas stated before: the better the conventional control performance, the better the LSTM-based one. For that reason, the DO LSTM-based PI is the one considered to perform the fine-tuning process. It is important to notice that the initial training of both structures, the DO LSTM-based PI and the NO LSTM-based PI is not compulsory. In Figure 4b it is clearly observed that the control loop offering the best performance corresponds to the PI managing the DO control loop. Hence, the DO LSTM-based PI can be initially adopted to be trained. Then, it will be transferred into the NO control loop and fined-tuned. As a consequence, there is no need to train or even implement the NO LSTM-based PI.

### 4.4. LSTM-Based Controller Fine-Tuning & Transfer

Once the control performance of the DO and NO LSTM-based PI controllers is computed one can clearly observe that the DO LSTM-based PI is the controller offering the best control performance in both control loops. For that reason, the fine-tuning of the DO LSTM-based PI controller is proposed. To perform this tasks, data coming from the default $S_{NO,2}$ PI controller is considered. In that sense, information about how to control and manage the $S_{NO,2}$ concentration is provided to the DO LSTM-based PI. Thus, the fine-tuned version of the controller, the FTDO LSTM-based PI, should be able to improve a better control performance in terms of the $S_{NO,2}$ managing process.

Now, the prediction performance of the FTDO LSTM-based PI equals to a RMSE of 0.095 mg/L, a MAE of 0.067 mg/L, a MAPE of 6.24% and a $R^{2}$ of 0.991. Its training time equals to 20.27 s. At first sight one can observe that prediction performance is degraded with respect to the DO and NO LSTM-based PI controllers. However, this degradation is motivated by the fact that the proposed FTDO LSTM-based PI controller has learnt how to correctly manage the $S_{O,5}$ and $S_{NO,2}$ concentration instead of a unique one. In addition, the training time in this occasion equals to 20.27 s, which means that the time spent in the fine-tuning process is largely reduced with respect to training the LSTM-based structure from scratch. This effect is motivated by the information already present in the LSTM structure, that is, the weights and biases of the blocked LSTM cells. This corroborates that TL techniques can be adopted to simplify and speed up the control design process. Let’s suppose that instead of transferring the knowledge of the DO LSTM-based PI into the NO control loop and performing a fine-tuning process, we decide to control each loop with its corresponding LSTM-based PI structure. The amount of time devoted to training the networks correspond to 69.91 and 98.60 s for the DO and NO control loops, respectively. This equals to a total time of 168.51 s only in terms of the training time. Although this time is affordable, if the DO LSTM-based PI is transferred into the NO control loop, only 69.91 s plus the time spent in the fine-tuning process, no more than 21 s is required. Thus, the total amount of time invested in the design process equals to 90.18 s, which represents a reduction of 78.33 s with respect to training two individual nets. Therefore, the reduction of the training time is clearly observed. In addition, it is important to notice that the WWTP we are dealing with only considers two control loops. However, this reduction of time will be higher in these situations where the number of control loops to design is larger. In that sense, an estimation of the training time reduction can be performed. If we suppose that the training time of the baseline LSTM-based PIs (from scratch) correspond to $t_{baseline}$ and that the time spent in the fine-tuning process on average equals to $t_{ft}$, the reduction of time (Δt) provided by our approach can be computed as:

(9) $$\Delta t=N\cdot t_{baseline}-\left[t_{baseline}+\left(N-1\right)\cdot t_{ft}\right]=\left(N-1\right)\left[t_{baseline}-t_{ft}\right],$$

where $t_{ft}\ll t_{baseline}$. *N* equals to the number of control loops where the baseline LSTM-based PI is the transfer. As it is observed, the higher the number of control loops to design, the higher the reduction of time and the higher the benefit of the proposed methodology. Not only this, but this methodology can also be applied in those situations where the control of a new WWTP scenario has to be designed. In such a context, the new control structure can be derived by transferring the knowledge of the control structure of an already controlled WWTP. This would involve an even higher reduction of the complexity and time required in the development of the control strategy. All these facts motivate us to consider the TL methods in the design of the WWTP control loops.

In terms of the control performance, results of the FTDO LSTM-based PI control are shown in Table 4, where the *IAE* and *ISE* values are computed for different weather profiles and set-points. It is worth noticing that the $S_{NO,2}$ is now managed by the fine-tuned and transferred DO LSTM-based PI, that is, the FTDO LSTM-based PI, whereas the $S_{O,5}$ concentration is managed by the DO LSTM-based PI.

**Figure 9:**
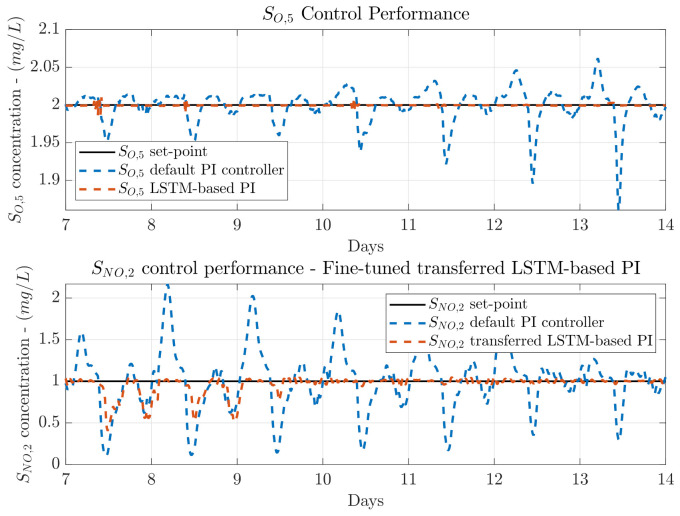
Control performance for the NO and DO control loops when a $S_{O,5}$ fix set-point and dry weather are considered. The LSTM-based PI managing the NO loop is transferred from the DO control loop and fined-tuned with data from the NO control loop.

When a fixed set-point is considered, one can observe that the control performance is hugely improved not only in terms of the $S_{O,5}$, but also in terms of the $S_{NO,2}$. The improvement of the DO control loop with respect to the default PI controller is translated into an average reduction of the *IAE* around a 95.94% and an average reduction in the *ISE* around a 99.78%. Thereby, this is translated in a better tracking process of the $S_{O,5}$ and consequently, a better management of this concentration. In terms of the NO control loop, one can observe that the *IAE* and *ISE* are hugely improved as well. However, there is an exception with the rainy weather. In this case, the $S_{NO,2}$ *IAE* and *ISE* are only improved a 40.17% and a 34.27%, respectively. This is motivated by the fact that the rainy weather profile shows two large perturbation during days 9 and 11. Besides, the fine-tuning process is performed with measurements obtained from the $S_{NO,2}$ default PI controller when a whole year of randomly distributed weathers is simulated. Thus, this entails that most of the knowledge provided to the DO LSTM-based PI consists in the control actuations to manage the $S_{NO,2}$ concentration when the dry weather is considered (remember that rainy and stormy weathers are equal to the dry weather with the exception of the two rainy and the two stormy episodes). On average, the NO control loop *IAE* and *ISE* are reduced by 73.47% and 72.84% with respect to the default $S_{NO,2}$ PI control performance. The greatest improvement is observed when the dry weather is considered. The *IAE* is reduced from 1.594 to 0.091, whereas the *ISE* is decreased from 0.691 to 0.002 (see Figure 9). In the case of the rainy weather, the reduction of the *IAE* and *ISE* is lower, the *IAE* changes from 1.922 to 1.150 and the *ISE* from 0.951 to 0.625. Nevertheless, this *IAE* value corresponds to the lowest NO control loop *IAE* value of the three TL approaches considered in this work.

**Figure 10:**
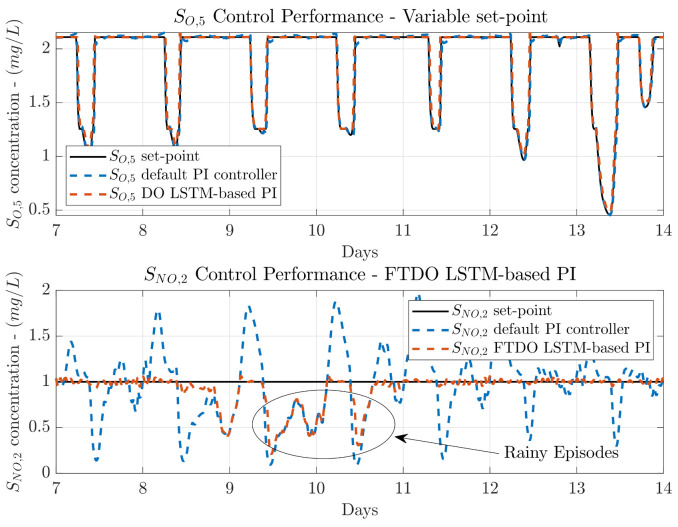
Control performance for the NO and DO control loops when a $S_{O,5}$ variable set-point and rainy weathers are considered. The LSTM-based PI managing the NO loop is transferred from the DO control loop and fined-tuned with data from the NO control loop.

Results of the control performance when a variable set-point is considered show the same tendency as the fix set-point ones. The *IAE* and *ISE* metrics have been improved for all the weather profiles. Again, the most important results are the ones corresponding to the NO control loop, which is the controller whose control performance improvement is sought with the fine-tuning process. In that sense, the best improvement is now observed when the dry weather is simulated. The *IAE* has been decreased from 1.792 to 0.129, which equals to an improvement of 92.80%. In terms of the *ISE*, it is decreased from 0.858 to 0.004, which represent an improvement of a 99.53%. It is important to notice that the lowest control performance is obtained when the rainy weather is considered, the *IAE* deceases from 2.132 to 0.643 while the *ISE* is reduced from 1.089 to 0.261. Although these improvements are not so high as the ones achieved with the dry weather, they are still much better than the performance obtained when the fine-tuning process is not carried out. For instance, the *IAE* has been improved a 69.84% whilst the *ISE* has been improved a 76.03%. The *IAE* improvement represents an increase of 48.26 and 42.63 percentage points with respect to the improvements achieved in the Transfer Learning from DO to NO and from NO to DO. In terms of the *ISE*, these increments equal to 45.64 and 36.82 percentage points, respectively. Visually, we can observe in Figure 10 that the $S_{NO,2}$ desired value of 1 mg/L is obtained at the same time the $S_{O,5}$ variable set-point is correctly tracked. In addition, the rainy episodes are plotted to show that the FTDO LSTM-based PI controller requires some more knowledge to finally learn how to manage these events.

As a summary, the control performance is improved in all terms regardless of the set-point topology and the weather profiles. This entail that the best option to design or improve a control strategy of an industrial plant, and especially a WWTP, is to obtain a first baseline controller, the DO LSTM-based PI, and then transfer its knowledge to the rest of control loops. The main point is to design the baseline controller with data coming from the controller performing better. In our case, this controller corresponds to the $S_{O,5}$ default PI controller. Then, the obtained DO LSTM-based PI is transferred into the remaining control loops and fine-tuned with data coming from controllers actuating in the target domain. Moreover, this approach entail that control loops can be designed without requiring a high knowledge of the different processes carried out in the plant. Only input and output measurements of a control strategy performing well are required. The rest of the control loops will be derived from the implemented one. Thus, the higher the number of control loops, the higher the benefit offered with this design approach. In our case, this benefit is not widely exploded since we have only transferred the LSTM-based PI between two control loops. However, this approach can be adopted in other scenarios where the number of control loops is largely higher than the ones managed here. In that sense, the benefit of this approach should be much higher than the one observed here.

Finally, the results observed in this manuscript motivates us to open a new research line where the transfer learning approach presented here is considered as the initial process of a reinforcement learning based control design. Then, instead of performing the fine-tuning process with measurement coming from a conventional control approach, it could be performed following a reinforcement leaning process. In that sense, the controller would be fine-tuned over time, adapting its output to the incoming measurements.

## 5. Conclusions

In this work, we presented a new industrial control design process which involves the application of LSTM-based neural networks and transfer learning approaches. The main purpose was to design and implement the control loops managing a general-purpose WWTP. The application is specific; however, the design approach can be adopted in any kind of industrial environment. The main idea is that TL techniques allow us to derive new control strategies from a baseline one without performing a deep tuning process of each control structure. This reduces the control design complexity, as well as the time invested in the training process of each data-based control structure. Thus, the higher the number of control loops, the higher the improvement achieved.

In our case, the proposed control design approach consists in two main processes: (i) the design of a neural network-based controller with data obtained from an existing control loop and (ii) the transfer of the controller knowledge into the remaining loops. To achieve that, three different design approaches were proposed, two of them mainly consisting in the design of the LSTM-based controller with data from a control loop and then transferring it to the others without retraining the net structure. In that sense, we considered the development of the LSTM-based PI controller either with measurements from the $S_{O,5}$ control loop or from the $S_{NO,2}$ one, both from a general-purpose WWTP. The third option considers the development of the LSTM-based PI with data from the $S_{O,5}$ control loop and the fine-tuning of its transferred version. Results show that there exists a trade-off between deriving the LSTM-based PI with measurement from the $S_{NO,2}$ or the $S_{O,5}$ control loops. If the LSTM-based PI controller is derived with measurements from the $S_{O,5}$ control loop, one can observe that the $S_{O,5}$ control performance is highly improved with respect to the default PI controller regardless of the weather influent and the considered set-point. In addition, the $S_{NO,2}$ control performance experiences a slight improvement as well. On the other hand, the NO control performance experienced an improvement at expense of degrading the $S_{O,5}$ control performance when the LSTM-based PI transferred into the DO control loop is implemented with measurements from the NO control loop. To solve this trade-off, we considered the third option, where the LSTM-based PI derived from the $S_{O,5}$ control loop was adopted and transferred into the DO control loop. Its transferred version was fine-tuned with measurements coming from the default PI controller managing the $S_{NO,2}$ control loop.

Results show that a high improvement is achieved in the $S_{O,5}$ control loop as well as in the $S_{NO,2}$ one. Besides, the lowest *IAE* and *ISE* improvements in terms of the $S_{NO,2}$ when compared to the default $S_{NO,2}$ PI controller equalled to 69.84% and 76.03% for the *IAE* and *ISE*, respectively, which are even higher improvements than in the cases where the fine-tuning process is not considered. This clearly shows that designing a LSTM-based PI in a control loop, transferring it to another different one, and then performing a fine-tuning process is the best option if a high level of improvement of the control performance is sought. Besides, this also entails a speed-up and a complexity reduction of the control design process since only the design and training of one control loop has to be performed. Again, the higher the number of control loops to design, the higher the benefit obtained following this design approach.

## Figures and Tables

**Table 1:** Prediction performance of the DO LSTM-based PI and the NO LSTM-based PI.

LSTM-Based Prediction Performance					
	**RMSE**	**MAE**	**MAPE**	$R^{2}$	**Training Time**
DO LSTM-based PI	0.026 mg/L	0.018 mg/L	1.347%	0.999	69.91 s
NO LSTM-based PI	0.048 mg/L	0.037 mg/L	6.26%	0.997	98.60 s

**Table 2:** Control performance when the DO LSTM-based PI derived from the DO control loop is transferred into the NO control loop.

Transfer Learning from DO to NO Control Loop						
Fix Set-point						
	Dry Weather		Rainy Weather		Stormy Weather	
Structure	*IAE*	*ISE*	*IAE*	*ISE*	*IAE*	*ISE*
PI—$S_{O,5}$	0.148	0.007	0.143	0.007	0.158	0.007
DO LSTM-based PI—$S_{O,5}$	0.006	$9.98\times 10^{-6}$	0.006	$1.12\times 10^{-5}$	0.006	$1.29\times 10^{-5}$
PI—$S_{NO,2}$	1.594	0.691	1.922	0.951	1.874	0.977
DO LSTM-based PI—$S_{NO,2}$	1.008	0.290	1.401	0.578	1.033	0.357
Variable Set-point						
PI—$S_{O,5}$	0.185	0.016	0.155	0.014	0.206	0.020
DO LSTM-based PI—$S_{O,5}$	0.013	$2.34\times 10^{-4}$	0.016	$4.48\times 10^{-4}$	0.016	$4.05\times 10^{-4}$
PI—$S_{NO,2}$	1.792	0.858	2.132	1.089	1.884	0.989
DO LSTM-based PI—$S_{NO,2}$	1.271	0.503	1.672	0.758	1.358	0.593

**Table 3:** Control performance when the NO LSTM-based PI derived from the NO control loop is transferred into the DO control loop.

Transfer Learning from NO to DO Control Loop						
Fix Set-point						
	Dry Weather		Rainy Weather		Stormy Weather	
Structure	*IAE*	*ISE*	*IAE*	*ISE*	*IAE*	*ISE*
PI—$S_{NO,2}$	1.594	0.691	1.922	0.951	1.874	0.977
NO LSTM-based PI—$S_{NO,2}$	1.302	0.486	1.399	0.542	1.360	0.543
PI—$S_{O,5}$	0.148	0.007	0.143	0.007	0.158	0.007
NO LSTM-based PI—$S_{O,5}$	0.158	0.004	0.146	0.004	0.160	0.004
Variable Set-point						
PI—$S_{NO,2}$	1.792	0.858	2.132	1.089	1.884	0.989
NO LSTM-based PI—$S_{NO,2}$	1.266	0.464	1.574	0.662	1.372	0.557
PI—$S_{O,5}$	0.185	0.016	0.155	0.014	0.206	0.020
NO LSTM-based PI—$S_{O,5}$	0.288	0.030	0.239	0.022	0.385	0.049

**Table 4:** Control performance when the DO LSTM-based PI derived from the DO control loop is transferred into the NO control loop. Then, the NO controller is fine-tuned with data from the default PI controller managing the $S_{NO,2}$.

LSTM-Based Controller Fine-Tuning & Transfer						
Fix Set-point						
	Dry Weather		Rainy Weather		Stormy Weather	
Structure	*IAE*	*ISE*	*IAE*	*ISE*	*IAE*	*ISE*
PI—$S_{O,5}$	0.143	0.007	0.143	0.007	0.158	0.007
DO LSTM-based PI—$S_{O,5}$	0.004	$5.12\times 10^{-6}$	0.008	$2.43\times 10^{-5}$	0.006	$1.76\times 10^{-5}$
PI—$S_{NO,2}$	1.594	0.691	1.922	0.951	1.874	0.977
FTDO LSTM-based PI—$S_{NO,2}$	0.091	0.002	1.150	0.625	0.357	0.151
Variable Set-point						
PI—$S_{O,5}$	0.185	0.016	0.155	0.014	0.206	0.020
DO LSTM-based PI—$S_{O,5}$	0.013	$1.99\times 10^{-4}$	0.017	$3.91\times 10^{-4}$	0.017	$3.72\times 10^{-4}$
PI—$S_{NO,2}$	1.792	0.858	2.132	1.089	1.884	0.989
FTDO LSTM-based PI—$S_{NO,2}$	0.129	0.004	0.643	0.261	0.324	0.122

## Data Availability

The data considered in this study correspond to the influent and effluent data generated and available in the same BSM1 framework defined in [8,25].

## Abbreviations

The following abbreviations are used in this manuscript:

ANN	Artificial Neural Network
ASM1	Activated Sludge Model N.1
BSM1	Benchmark Simulation Model No. 1
BSM1-P	Benchmark Simulation Model No. 1 with Phosphorus processing
BSM2	Benchmark Simulation Model No. 2
$b_{x}$	Biases of the *x*th hidden layer
DO	Dissolved Oxygen in the fifth reactor tank ($S_{O,5}$) control loop
DO LSTM-based PI	LSTM-based PI controller trained with data from the $S_{O,5}$ control loop
FTDO LSTM-based PI	DO LSTM-based PI fine-tuned with data from the $S_{NO,2}$ control loop
*IAE*	Integrated Absolute Error
*ISE*	Integrated Squared Error
$K_{La,x}$	Oxygen Transfer Coefficient of the *x*th reactor tank measured in ${\mathrm{day}}^{-1}$
LSTM	Long Short-Term Memory
MAE	Mean Absolute Error
MAPE	Mean Average Percentage Error
MLP	Multilayer Perceptron
MPC	Model Predictive Controller
NO	Nitrate and nitrite nitrogen in the second reactor tank ($S_{NO,2}$) control loop
NO LSTM-based PI	LSTM-based PI controller trained with data from the $S_{NO,2}$ control loop
PI	Proportional Integral controller
PID	Proportional Integral Derivative controller
$Q_{0}$	Influent flow rate
$Q_{a}$	Internal recirculation flow rate
$Q_{r}$	External recirculation flow rate
$R^{2}$	Determination coefficient
RMSE	Root Mean Squared Error
$S_{NO,x}$	Nitrate and nitrite nitrogen in the *x*th reactor tank measured in mg/L
$S_{NH,x}$	Ammonium concentration in the *x*th reactor tank measured in mg/L
$S_{O,x}$	Dissolved oxygen concentration in the *x*th reactor tank measured in mg/L
TL	Transfer Learning
$U_{x}$	Weights affecting the previous output data of the *x*th hidden layer
WWTP	Wastewater Treatment Plant
$W_{x}$	Weights affecting the input data of the *x*th hidden layer
